# Maternal epileptic seizure induced by Pentylenetetrazol: Apoptotic neurodegeneration and decreased GABA_B1 _receptor expression in prenatal rat brain

**DOI:** 10.1186/1756-6606-2-20

**Published:** 2009-06-22

**Authors:** Muhammad Imran Naseer, Li Shupeng, Myeong Ok Kim

**Affiliations:** 1 Division of Life Science, College of Natural Sciences and Applied Life Science (Brain Korea 21), Gyeongsang National University, Chinju, 660-701, Republic of Korea

## Abstract

Epilepsy is a prominent sign of neurological dysfunction in children with various fetal and maternal deficiencies. However, the detailed mechanism and influences underlying epileptic disorders are still unrevealed. The hippocampal neurons are vulnerable to epilepsy-induced pathologic changes and often manifests as neuronal death. The present study was designed to investigate the effect of maternal epileptic seizure on apoptotic neuronal death, modulation of GABA_B1 _receptor (R), and protein kinase A-α (PKA) in prenatal rat hippocampal neurons at gestational days (GD) 17.5. Seizure was induced in pregnant rat using intraperitoneal injection of pentylenetetrazol (PTZ) (40 mg/kg for 15 days). To confirm the seizure electroencephalography (EEG) data was obtained by the Laxtha EEG-monitoring device in the EEG recording room and EEG were monitored 5 min and 15 min after PTZ injection. The RT-PCR and Western blot results showed significant increased expression of cytochrome-c and caspases-3, while decreased levels of GABA_B1_R, and PKA protein expression upon ethanol, PTZ and ethanol plus PTZ exposure in primary neuronal cells cultured from PTZ-induced seizure model as compare to non-PTZ treated maternal group. Apoptotic neurodegeneration was further confirmed with Fluoro-Jade B and propidium iodide staining, where neurons were scattered and shrunken, with markedly condensed nuclei in PTZ treated group compared with control. This study for the first time indicate that PTZ-induced seizures triggered activation of caspases-3 to induce widespread apoptotic neuronal death and decreased GABA_B1_R expression in hippocampal neurons, providing a possible mechanistic link between maternal epilepsy induced neurodegeneration alteration of GABA_B1_R and PKA expression level during prenatal brain development. This revealed new aspects of PTZ and ethanol's modulation on GABA_B1_R, learning and memory. Further, explain the possibility that children delivered by epileptic mothers may have higher risk of developmental disturbances and malformations.

## Background

It is well established that the development of an organism is not only determined by genetic, and postnatal environment effects, but also by prenatal effects e.g. during gestation. Modifications of various neurotransmitter systems and neuronal excitability can be induced at early stages of development by behavioural procedures and by prenatal exposure of various substances [1-3]. In clinical medicine it is well established that children delivered by epileptic mothers may have a higher risk of developmental disturbances and malformations [4].

Epilepsy is one of the most prevalent neurological disorders with current estimates approximating between 0.5–2% of the global population being affected. Epileptic convulsions have significant influences on brain structure and are able to induce neuron death. The earliest morphological changes associated with prolonged convulsive activity consist of selective cell death in epileptogenic structures, primarily the hippocampus [5]. Although the detailed molecular mechanisms are still under investigation, present physiological and genetic analysis reveal that epilepsy is closely related with the various ion channels including voltage-gated channels (Na+, K+, Ca2+, Cl-) and ligandgated channels (nicotinic acetylcholine and GABA_A _receptors).

Apoptosis is a normal process in the developing brain; for optimal development, greater than 50% of the original neurons must undergo programmed cell death or apoptosis [6]. Mitochondria play an important role in apoptosis under a variety of proapoptotic conditions, such as oxidative stress [7]. Mitochondrial cytochrome-c release is a key event in the activation of caspase-3, a downstream pivotal step to initiate apoptosis [8]. Neurodegeneration exhibited as reduced brain mass and neurobehavioral disturbances in many neurological disorders including epilepsy and fetal alcohol syndrome (FAS). The cell death appears to be associated with activation of caspases-3, an executioner protease that is activated during apoptosis cell death [9,10].

GABA_B _receptor (R) is known to play an important role during the development of central nervous system (CNS) and the role of GABA_B_R in epilepsy has been demonstrated in genetic models of absence seizures in rodents [11-13]. Molecular expression studies and gene deletion experiments provide unequivocal evidence for modifications of GABA_B1_R subunits in the development of seizures, hyperalgesia, hypothermia, memory impairment, anxiety and retarded growth all of which provide important clues about the role of GABA_B1_R in controlling brain function [14-17]. GABA_B1_R agonists promote and antagonists inhibit convulsive activity in these models and GABA_B1_R appear to be functionally up regulated in epileptic mice [18-21]. Pentylenetetrazol (PTZ) is a blocker of the chloride ionophore complex to the GABA_A _receptor [22] that has convulsant effects after repeated or single-dose administration and also affects several neurotransmitter systems, such as the GABAergic and glutamatergic systems [23-26]. Both GABA_A _and GABA_B_Rs are involved in the control of neuronal excitability and epileptogenesis but, whereas much is known about the involvement of GABA_A_Rs in the control of generalized convulsive seizures [27]. However, little is known about the role of GABA_B1_R in epilepsy and its possible molecular expression and distribution.

The aim of present study was to examine the neuronal apoptotic and morphological changes in the hippocampal neurons of prenatal rat following PTZ-induced seizure during pregnancy and its relation with the expression of GABA_B_R as well as PKA protein level. Our results revealed that PTZ-induced seizure cause apoptotic neurodegeneration, and decreased GABA_B1_R expression which further leads to intracellular changes at PKA expression level. These results provide first molecular evidence of apoptotic neurodegeneration and decreased GABA_B1_R expression in developing brain due to PTZ-induced seizure during pregnancy.

## Results

### Seizure behavior and EEGs during seizures

**Figure 1:**
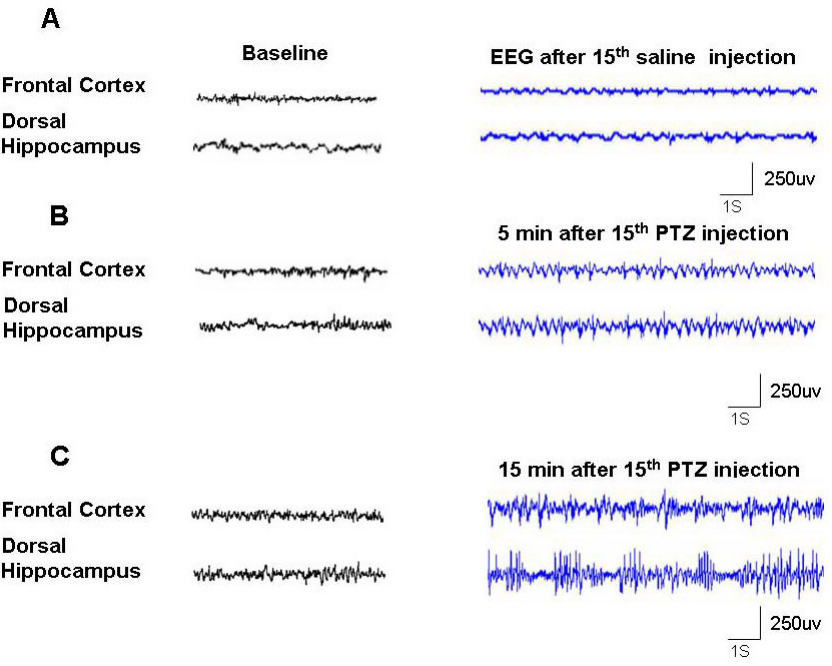
**Representative EEG traces in pregnant rat after 15^th ^injections**. Representative 30 s EEG samples recorded 5 and 15 min after 15^th ^injection of PTZ (40 mg/kg) during pregnancy at day 16^th^. **A**; Showed normal EEG in saline group **B; **EEG 5 min after 15^th ^PTZ injection and **C; **EEG 15 min after 15^th ^PTZ injection.

Chronic administration for a period of 15 days with a sub convulsive dose of 40 mg/kg during pregnancy induced minimal seizures corresponding to score 3–4. In addition, barrel rotations and tonic seizures were noticed in some of the animals: 65% of the animals at the 10^th ^injection up to 85% at the 15^th ^injections. A seizure induced by PTZ in pregnant rat typically started with hind limb kicks, followed by generalized tonic and clonic convulsion of four limbs while laying down. The results of acute observations after PTZ injections are summarized in (Table 1). To confirm the seizure, EEG was monitored for 30 min started 5 and 15 min after the PTZ injection. Five rats from the control group and 15 min from the PTZ treated group were monitored. The results for the evaluation of EEG monitoring are summarized in figure 1.

**Table 1:** Acute observations after PTZ injection

	PTZ seizures
Number of seizures	2.1 ± 1.5
Latent period (s)	60 ± 21.5
Seizure time (min)	4.8 ± 0.9
Unilateral or bilateral forlimb jerks	1.9 ± 0.5
Mouth automatisms	5.1 ± 0.7
Rearing with bilateral forlimb jerks	3.1 ± 0.9
Fall or lying down with four limbs jerks	4.5 ± 0.4

Number of seizures showing a type of behavior during seizure induced by PTZ in pregnant rats after 15^th ^injection, all the data in the table is presented in mean ± standard deviation (SD).*p < 0.05.

### PTZ-induced seizure enhances the release of cytochrome-c

**Figure 2:**
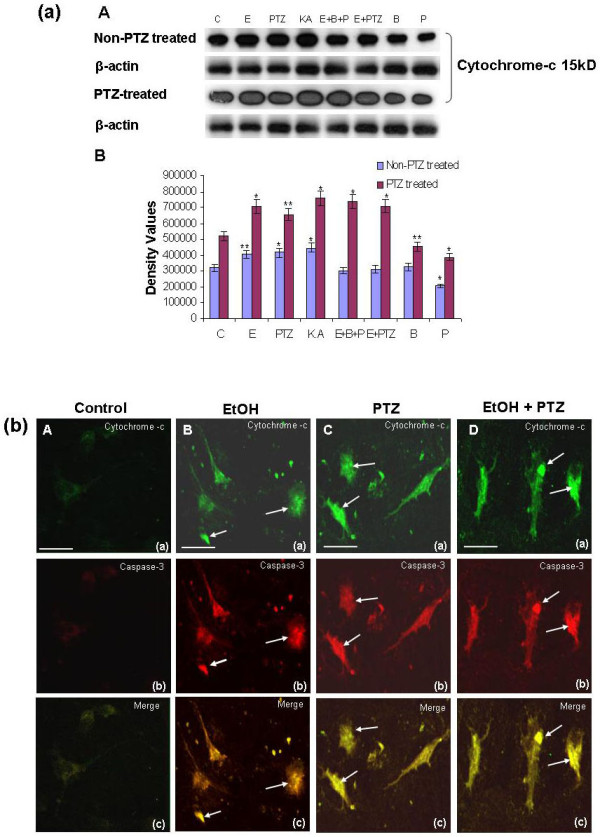
**(a) PTZ induced seizure increases the release of cytochrome-c from mitochondria in prenatal rat hippocampal neurons**. Western blot analyses (**2a**) of the cytochrome-c in the primary cultured hippocampal neuronal cells at GD 17.5 from PTZ-induced seizure model during pregnancy. Cells were treated for 20 min with normal media as control (C), media containing 100 mM ethanol (E), media contain 10 mM pentylenetetrazol (PTZ), media contain 1 μM Kainic acid (KA), media contain PTZ and ethanol (E+PTZ), media contain ethanol plus baclofen plus phaclofen (E+B+P), media contain 50 μM baclofen (B), and media contain 100 μM phaclofen (P) respectively. β-actin is taken as loading control in each case. **A**: Immunoblots of cytochrome-c of hippocampal neuronal cells under different treatment conditions. The immunoblots were labeled with an anti cytochrome-c antibody. **B**: Density values were expressed as mean ± SEM (*n *= 4, mean four rat per group) of the corresponding protein of cytochrome-c are presented. The density values on (Y-axis) are expressed as arbitrary units (AU). *P < 0.05 and **P < 0.01 versus control group. **(b) Visualization of mitochondrial cytochrome c release and caspase-3 expression in same neuronal cells**. In situ analysis of cytochrome-c release and caspase-3 expression was carried out by immunofluorescence technique upon exposure of ethanol and PTZ in hippocampal neuronal cells culture. Primary neuronal cells culture of prenatal rat exposed to PTZ and ethanol for 20 min. Detail procedures are mentioned in materials and methods section. The immunofluorescence of hippocampal cell double stained for cytochrome-c (FITC-labeled cytochrome-c antibody, green) and caspase-3 (TRITC-labeled, red). Yellow color (green + red; merge image) indicates release of cytochrome-c from mitochondria into cytosol and caspase-3 in the same neuronal cells, as detected by confocal microscopy. Arrow indicates the dead cell with release of cytochrome-c and caspse-3 expression. Magnification 40×, Scale bar = 20 μm for A-C.

Cytochrome-c is a mitochondrial inner membrane protein, which upon release into the cytosol elicits a cascade of events that ultimately activates caspases-3. To study the effect of PTZ-induced seizure during pregnancy, prenatal rat hippocampal neurons were treated with ethanol, PTZ, kainic acid (KA 1 μM), baclofen (50 μM) and phaclofen (100 μM) for 20 min with different combination. The release of cytochrome-c was examined with Western blot analysis. Figure 2(a) illustrated that exposure of cultured hippocampal neurons to ethanol, PTZ, ethanol plus PTZ and KA caused significant increased expression of cytochrome-c in PTZ-induced seizure as compare to non PTZ treated group, suggesting that release of cytochrome-c into the cytoplasm induces the formation of apoptosome. These observations were further confirmed in primary hippocampal neuronal cells culture by the translocation of cytochrome-c and caspase-3 using confocal microscopy which revealed a diffuse staining pattern of cytochrome-c, suggesting its release from mitochondria (cytochrome-c, green FITC-labeled) along with caspase-3 (TRITC-labeled, red) upon treatments with PTZ, ethanol and PTZ plus ethanol. The merge image (yellow) indicated the release of cytochrome-c and caspase-3 expression into cytosol as a result of PTZ and ethanol exposure in the hippocampal neuronal cells culture (Figure 2b).

### Caspase-3 activities increased in prenatal rat hippocampal neurons

**Figure 3:**
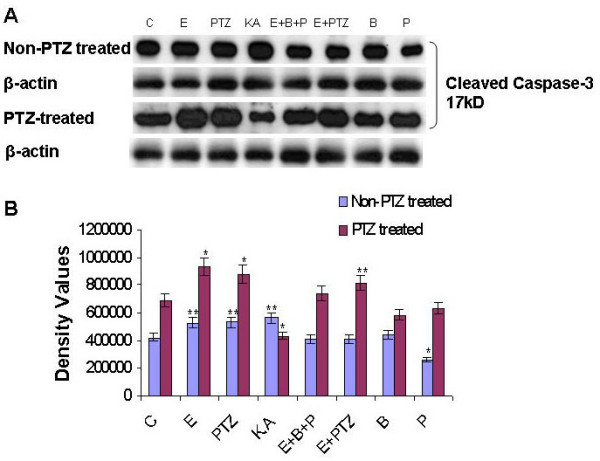
**PTZ induced seizure increased the expression of cleaved caspases-3 in prenatal rat hippocampal neurons**. Western blot analyses of the caspases-3 in the primary cultured hippocampal neuronal cells at GD 17.5 from PTZ-induced seizure model during pregnancy. Cells are exposed to different drugs for 20 min as previously explained. Immunoblotting with polyclonal antibody showed processing of caspases-3 with the appearance of active fragment (17 kDa cleaved caspase-3). Detail procedures are mentioned in material and method section. β-actin is taken as loading control in each case. **A**: Immunoblots of caspases-3 of hippocampal neuronal cells under different treatment conditions. The immunoblots were labeled with an anti caspases-3 antibody **B**: Density values were expressed as mean ± SEM (*n *= 4, mean four rat per group) of the corresponding protein of caspases-3 are presented. The density values on (Y-axis) are expressed as arbitrary units (AU). *P < 0.05 and **P < 0.01 versus control group.

Release of cytochrome-c could in turn activate caspases-3 [9,28]. In order to determine whether PTZ-induced seizure cause activation of caspases-3, we examined expression changes of caspases-3 in hippocampal neurons during early developmental stage using Western blot analysis. Caspase-3 in physiological conditions exists as a 32-kDa procaspase and when activated, is cleaved into a small prodomain and two subunits of 17 and 12 kDa, respectively. We found that PTZ-induced seizure and *in vitro *exposure of ethanol, PTZ and ethanol plus PTZ caused significantly increased expression of cleaved caspases-3 in prenatal rat hippocampal neurons as compare to control group (Figure 3). Taken together, these findings indicate that the proteolytic cleavage of caspase-3 into active caspase-3 fragments is induced by PTZ and ethanol exposure during pregnancy.

### PTZ-induced seizure causes apoptotic neurodegeneration

**Figure 4:**
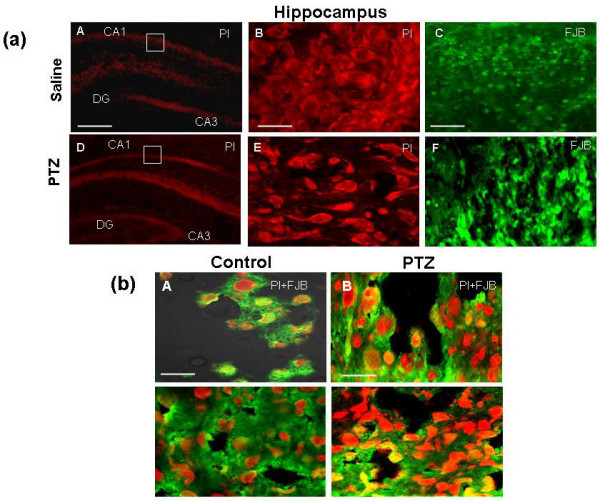
**PTZ-induced seizure induced apoptotic neurodegeneration**. **(a)**: Propidium iodide (PI) and Fluoro-jade B staining analysis of the neurodegeneration in prenatal rat hippocampal from PTZ-induced seizure models. Detail procedures are mentioned in material and method section. These histological sections are from prenatal rat at GD 17.5 days-old to a vehicle-treated mother. Maternal exposure of PTZ during pregnancy induces neurodegeneration in the hippocampal area of fetal rat brain (A-F). Interruptions in the CA1 lamination, clear neurodegeneration and change in cell morphology were observed in prenatal fetal of pregnant rat treated with PTZ. The photograph documented that in prenatal rat brain from PTZ-induced seizure model has triggered a robust neurodegeneration reaction throughout many regions of the fetal rat hippocampal areas, whereas saline has left the brain showing only a sparse of apoptotic degeneration attributable to physiological cell death that occurs normally in the developing brain. Panels (B, E = ×100 and C, F = ×40) are magnified views from panels (A, D = ×20), representing PI and FJB staining in hippocampal area. **(b)**: Representing the co treatment of FJB and PI staining in hippocampal area of brain. Magnification 100×, Scale bar = 10 μm.

To assess whether PTZ-induced seizure during pregnancy induced *in vivo *neuronal cell death in hippocampus, histological analysis with PI and Fluoro-Jade-B (FJB) staining was performed. PI is a nucleic acid satin usually used as a counter stain in multicolor fluorescence techniques. In tissue section, it is used as a nuclear marker and sometimes employed to identify nuclei showing apoptotic changes [29,30]. Confocal microscopic analysis revealed that there was a robust staining throughout the hippocampal area in prenatal rat brain from PTZ-induced seizure model as compare to control group. In hippocampal CA1 subfields, pyramidal neurons were scattered and shrunken, and the nuclei were markedly condensed (Figure 4a, A–F). PI and FJB staining showed neuronal cells with normal morphological properties exhibiting round nuclei in control group, but many cell begin to shrink and nuclei were condensed in treated group. Fluoro-Jade-B and PI staining, predominantly a marker of neuronal injury, revealed neurodegeneration throughout prenatal rat hippocampal area from PTZ-induced seizure model (Figure 4b), the co-treatment of Fluoro-Jade-B and PI staining at high magnification showed a dramatic increase in Fluoro-Jade-B and PI labeling in hippocampal area of prenatal rat form PTZ-induced seizure model as compare to saline treated group.

### PTZ-induced seizure modulate GABA_B1_R expression in prenatal rat hippocampal neurons

**Figure 5:**
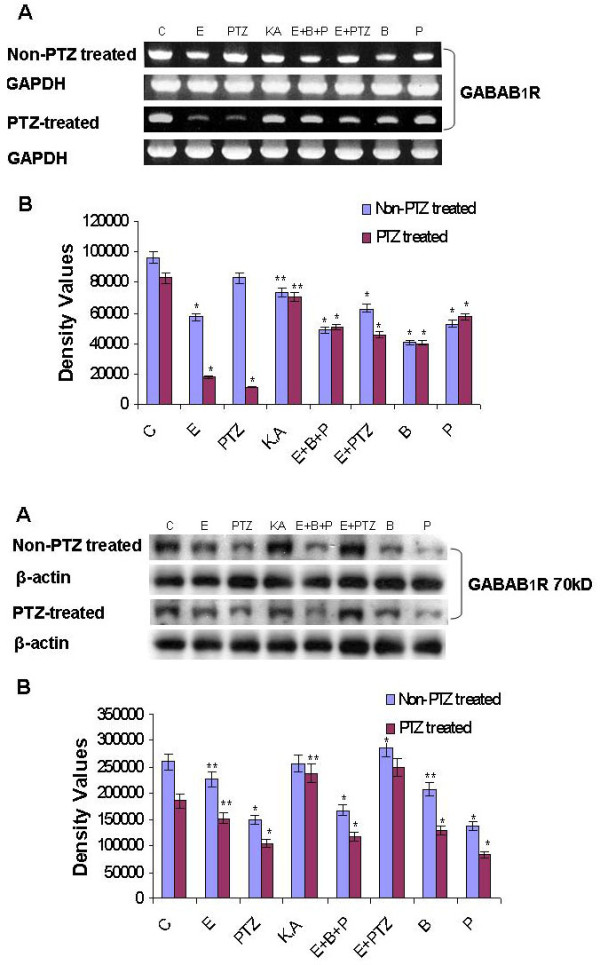
**(a, b) PTZ-induced seizure during pregnancy decreases the mRNA and protein level of GABA_B1_R in prenatal rat hippocampal neurons**. **A**: RT-PCR analyses change in mRNA (**5a**) and protein level (**5b**) of the GABA_B1_R in the primary cultured hippocampal neuronal cells at GD 17.5 from PTZ-induced seizure model during pregnancy. Cells are exposed to different drugs for 20 min as previously explained. Detail procedures are mentioned in material and method section. GAPDH and β-actin is taken as control. In case of Western blot analysis immunoblots were labeled with an anti GABA_B1_R antibody. **B**: Density values were expressed as mean ± SEM (*n *= 4, mean four rat per group) of the corresponding mRNA of GABA_B1_R are presented. The density values on (Y-axis) are expressed as arbitrary units (AU). *P < 0.05 and **P < 0.01 versus control group.

To further explore the *in vivo *changes of GABA_B1_R and its relation with PTZ-induced seizure effect on hippocampal neuronal cells, primary cultured cells from control, baclofen, phaclofen treatment were studied and GABA_B1_R mRNA level was examined by using RT-PCR. The results showed that GABA_B1_R expression at mRNA level in hippocampal neurons was decreased significantly in PTZ-induced seizure model as compare to control group (Figure 5a). We also examined Western blot analysis whether the protein changes resulted from the mRNA changes. The results showed that PTZ-induced seizure significantly decreased GABA_B1_R protein expression in hippocampal neurons (Figure 5b), whereas phaclofen (GABA_B_R antagonist) further decreased the GABA_B1_R expression. Thus, the decreased protein level of GABA_B1_R expression suggests that PTZ-induced seizure during pregnancy modulate the GABA_B1_R protein expression in the fetus of developing brain (Figure 5b).

### PTZ-induced seizure modulates PKA-α expression

**Figure 6:**
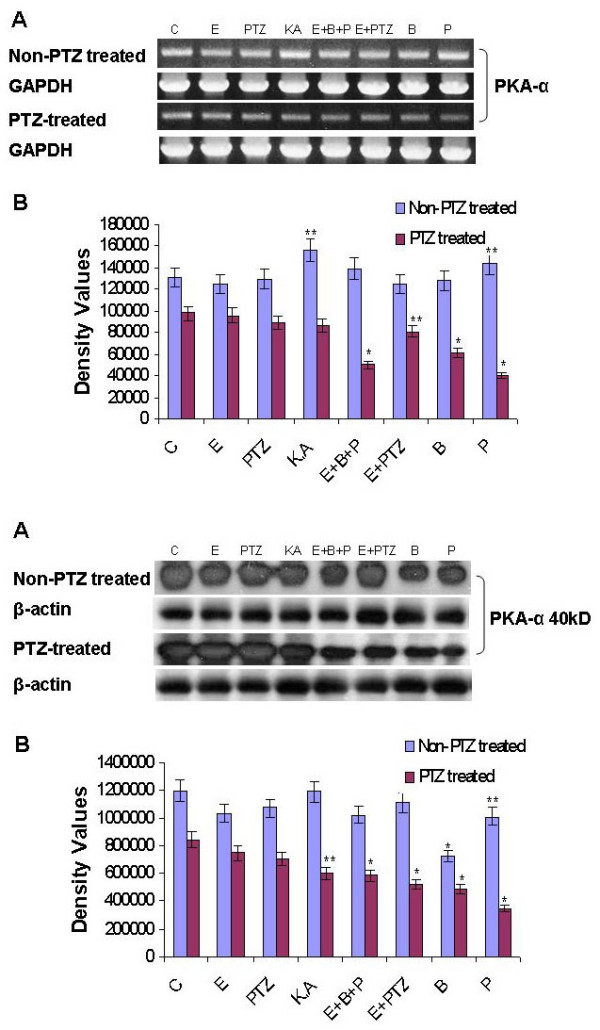
**(a, b) PTZ-induced seizure during pregnancy decreases the mRNA level of PKA-α in prenatal rat hippocampal neurons**. **A**: RT-PCR analyses change in mRNA (**6a**) and protein level (**6b**) of the PKA-α in the primary cultured hippocampal neuronal cells at GD 17.5 from PTZ-induced seizure model during pregnancy. Cells are exposed to different drugs for 20 min as previously explained. Detail procedures are mentioned in material and method section. GAPDH and β-actin is taken as control. In case of Western blot analysis immunoblots were labeled with an anti PKA antibody. **B**: Density values were expressed as mean ± SEM (*n *= 4, mean four rat per group) of the corresponding mRNA of GABA_B1_R and PKA are presented. The density values on (Y-axis) are expressed as arbitrary units (AU). *P < 0.05 and **P < 0.01 versus control group.

PKA is a major modulator of synaptic transmission likely to be involved in molecular and cellular events leading to epileptogenesis. PTZ administration during pregnancy could elicit various intracellular changes, including changes in the second messenger cAMP, which could further activate PKA to modulate downstream gene expression. To further explore the modulating effects of maternal epileptic seizure on the expression of PKA protein, we examined the PKA levels on PTZ treatment by using RT-PCR and Western blot analysis. The RT-PCR results showed that PKA expression was not decreased significantly upon ethanol, KA, ethanol plus PTZ, baclofen and phaclofen treatment in hippocampal neurons (Figure 6a), whereas Western blot showed significant decrease expression of PKA upon exposure of ethanol, PTZ, ethanol plus PTZ, KA, baclofen and phaclofen treatment both in PTZ treated and non PTZ treated groups, these results suggest that the effect of seizure induced by PTZ had direct effects on PKA expression level but could modulate PKA via the associated changes of GABA_B1_R expression levels (Figure 6b).

## Discussion

In present study, we have observed the effects of PTZ-induced seizure model on apoptotic neuronal death, GABA_B1_R and PKA expressional changes in prenatal rat hippocampal neurons. The in vitro effects of ethanol, PTZ, KA, baclofen and phaclofen were also observed. Our findings indicate that primary culture from PTZ-induced seizure model triggers robust caspase-3 activation, release of cytochrome-c and decrease GABA_B1_R expression which has further decreasing effect on PKA expression level in hippocampal neurons of prenatal rat brain, while the co treatment of baclofen and phaclofen reverse the effects of PTZ and provide neuroprotection.

It is well known that PTZ cause epileptic seizures and brain damage, acting on defined receptors groups and it is well documented that the consequences of status epilepticus in the developing brain differ from those of the mature brain [31-34]. Previously, it is reported that transient decreases in GABA_B_R mRNA expression in all subfields of the hippocampus after KA-induced seizures. This is best exemplified by the transient decrease in GABA_B_R mRNA levels in granule cells that are resistant to seizure-induced damage [35]. Since the early decreases in GABA_B_R mRNAs in CA1 and CA3 pyramidal cells essentially preceded the prominent seizure-induced cell losses seen in this animal model [35,36].

PTZ-induced seizure that elicited cell death in the brain of experimental prenatal rats was examined using an immunofluorescence, Western blot, propidium iodide and fluoro jade B for detection of neurodegeneration. Cytochrome-c is a mitochondrial inner membrane protein, which upon release into the cytosol, elicits a cascade of events that ultimately activates caspases. Caspases can be subdivided into several groups based on structure, function, and/or position in the apoptotic pathway [37]. One such scheme divides caspases into three subgroups, one involved in cytokine processing, a second that initiates the apoptotic cascade, and a third that represents the true effectors enzymes in apoptotic death. This third group consists of caspases 3, 6, and 7. Caspase-3 has been the focus of intense neuroscience investigations and appears to be the predominant effectors caspases in the developing nervous system [38,39]. Because the activated caspase-3 molecule is distributed widely throughout each affected neuron, and many types of neurons are affected by ethanol. This will provide valuable information at molecular level which may shed light on the neuropathlogical origins of the behavioral deficits that epilepsy victims display. The absence of apoptotic nuclei at neurodegeneration sites may result either from elimination of neurons dying from apoptosis at GD 17.5 after the end of PTZ kindling or from neuron death was being via a non-apoptotic pathway. In the former case, release of cytochrome-c and activation of caspase-3 might be a significant stage in the mechanism of apoptotic neurodegeneration.

PTZ-induced seizure is associated with an imbalance between excitatory and inhibitory neurotransmissions where long-term reduction of GABA-mediated inhibition in the cortex increases the seizure susceptibility [40,41]. PTZ produces proconflict and convulsant effects in rodents [42], but cognitive deficits have also been noted [43]. These pharmacological effects of PTZ appear to be mediated through a specific interaction with the GABA-gated chloride ionophore. Recent studies on the mechanisms involved in chemical kindling have shown that PTZ kindling is associated with a decrease in the biochemical indices of central GABAergic function [40]. Pre-synaptic GABA_B_Rs suppress release of both glutamate and GABA [44] and the effects on excitatory and inhibitory pre-synaptic terminals could change during ontogeny. Thus, the expressional changes of GABA_B1_R may underlie the molecular mechanism of PTZ-induced epilepsy.

PKA is a major modulator of synaptic transmission likely to be involved in molecular and cellular events leading to epileptogenesis. Previous results showed that acute picrotoxin-induced seizures occur without an increase in hippocampal PKA activity [45], but reduced PKA-mediated phosphorylation protects against picrotoxin seizures, probably by increasing the inhibitory potential of GABA(A) receptors. This is in accordance with our results that PTZ-induced seizure showed slightly decreased expression of PKA at mRNA levels, but showed significantly decreased expression at protein level, whereas baclofen and phaclofen could also modulate the PKA levels. Former results revealed that GABAergic inhibitory synaptic transmission are regulated by phosphorylation of GABA_A_Rs. Biochemical approaches demonstrated that GABA_A_Rs can be phosphorylated directly and consequently can be functionally modulated by PKA. The modulation of GABA_B _receptor upon PTZ-induced seizure and/or GABA_B_R agonist/antagonist could further induced the PKA changes via direct or indirect crosstalk [46-48].

## Conclusion

We conclude that PTZ-induced seizure induces apoptotic neurodegeneration and triggers a robust pattern of caspase-3 activation, release of cytochrome-c in primary culture of prenatal rat hippocampal neurons. PTZ-induced seizure decrease GABA_B1_R expression which has further decreasing effect on PKA expression level, which may provide an explanation for the reduced brain mass and neurobehavioral disturbance associated with seizure during early brain development and revealed new aspects of PTZ and ethanol's modulation on GABA_B1_R, learning and memory. Further, explain the possibility that children delivered by epileptic mothers may have higher risk of developmental disturbances and malformations.

## Methods

### Animal treatment

Female (*n *= 48) Sparague-Dawley rats (250 g, Gyeongsang National University, Neurobiology Laboratory, Chinju, South Korea) were housed in a temperature-controlled environment with lights from 06:00–20:00 h with food ad libitum. Timed pregnant [the day of insemination equals to GD 0.5]. After gestational days (GD) 17.5 pregnant Sparague-Dawley was killed by decapitation, after an i.v. injection of pentobarbital sodium (3 mg/100 g b.w).

The animals were randomly divided into two experimental groups:

(1) PTZ treated group: female rat which received PTZ injection (40 mg/kg) i.p from 2–16 days after insemination.

(2) Control: 0.9% saline solution was given i.p.

### Seizure observation procedures and EEG recording

Over a period of 17 days, animals were injected intraperitoneally with sub convulsive doses of PTZ (40 mg/kg) in saline every 24 h control group were given only saline injection. After each injection, the convulsive behavior was observed for 30 min, and resultant seizures were scored as follows: stage 0, no response; stage 1, ear and facial twitching; stage 2, convulsive waves axially through the body; stage 3, myoclonic jerks and rearing; stage 4, clonic convulsions with the animal falling on its side; and stage 5, repeated severe tonic-clonic convulsions or lethal convulsions. The animals were considered to be kindled after having received 10 PTZ injections and having reached at least three consecutive stage 4 or stage 5 seizures [49]. Latency was defined as the average length of time in minutes between drug administration and seizure onset. The generalized seizure was characterized by symmetric forelimb and hind limb tonus, and then hind limb clonus and flipping activity. Since an animal occasionally had another fit either while the first one was going on or somewhat later than the first one, the seizure duration was calculated as the sum of these multiple seizures for each animal to be assessed as one combined fit. The researcher injecting the rat and observing the seizure was blind to the exposure condition PTZ of each rat. Subsequently, latency to first seizure onset, total seizure duration, the number of seizure episodes recorded for each subject. EEG data were recorded for 30 min using amplifiers (LAXTHA, LXEJ 108) and were digitized at 250 Hz in EEG recording room. Whole EEG samples were analyzed by visual inspection for the presence of epileptiform activity as previously defined [50,51].

### Primary cell culture and drug treatment

Pregnant rats were given i.p injections of PTZ (40 mg/kg) daily between gestational days 2–16. Cultures were prepared from the hippocampal neurons of prenatal rat at GD 17.5 from pregnant rats. Pooled hippocampal tissues were treated with 0.25% trypsine-EDTA for 20 min and dissociated by mechanical trituration in ice-cold calcium- and magnesium-free Hank's balanced salt solution (pH 7.4). After pelleting by centrifugation, cells were plated (1 × 10^6 ^cells/ml) in cell culture plates pre-coated with poly-lysine (0.02 g/l) and chamber slides. The culture medium consisted of Dulbecco's modified Eagle medium (DMEM) with 10% heat-inactivated fetal bovine serum, 1 mM pyruvate, 4.2 mM sodium bicarbonate, 20 mM HEPES, 0.3 g/l bovine serum albumin, 50 U/ml penicillin, and 50 mg/l streptomycin. Cultures were maintained at 37°C in a humidified atmosphere of 5% CO_2 _and 95% air. Neuroglia cells were inhibited with media containing 100 μM Cytosine β-D-Arabino Furanoside (Sigma) for 12 h. After 3 days, hippocampal neuronal cells were treated with media contain ethanol 100 mM, PTZ 10 mM, baclofen 50 μM and phaclofen 100 μM in different groups and combinations. All drugs treated groups were incubated for 20 min *in vitro *culture.

### Reverse transcriptase-polymerase chain reaction (RT-PCR)

RT-PCR analysis was performed using cDNA from drug treated groups. Total RNA was isolated with Trizol Reagent (Life Technologies, Rockville, MD). First strand cDNA was transcribed from 2 μg of RNA using oligo (dt)_15_, M-MLV reverse transcriptase (Promega), following the protocol provided by the company. Total 4 μl of cDNA was used for PCR amplification in presence of 1 μl Taq DNA polymerase. Thermal cycling was performed under the following conditions: 94°C for (5 min), 30 cycles at 94°C (1 min) 68°C for (1 min), and 72°C for (1 min) followed by 72°C (5 min) for the final extension. As a negative control GAPDH (58°C, 25 cycle) was performed. PCR product were run on a 1% agarose gel containing ethidium bromide and viewed under UV light. The primers used were the following: GABA_B1_R forward primer 5'AATTGAATTCCGCTACCATCCAACAGACCA3'; GABA_B1_R reverse primer 5'AATTAAGCTTTCCTGTGACGTCATGTTGGAA3' PKA forward: 5'GTGGCAAGGAGTTTACTGAG3' PKA reverse: 5' CCAGTATCTGACTTTCCTGC 3' GAPDH forward: 5' GCCATCAATGACCCCTTCATT3' GAPDH reverse: 5'CGCCTGCTTCACCACCTTCTT3'.

### Western blotting

Primary cultured hippocampus cells were homogenized in cell lysis buffer (Cell signaling #9803) with protease inhibitor 100 mM PMSF. Sample was placed in ice for 20 min before sonication for 4 min (operate 15 sec, pause 10 sec). After ultra centrifugation (12,000 rpm, 10 min ×2), the protein contain supernatant was separated. The protein content was measured spectrophotometrically at 595 nm using the Bio-Rad Protein Assay and 30 μl protein was applied per lane. The soluble fraction (30 μg) was separated on duplicate 12% SDS-polyacrylamide gels (30%Acrylamide, 1% Bis, 1 M Tris, 10% SDS, 10% APS, TEMED). One gel was stained with Comassie Blue, while the proteins on the other gel were transferred onto a nitrocellulose membrane (90 V for 1 h in a 48 mM Tris, 39 mM glycine, 20% MeOH and 0.037% SDS transfer buffer). The nitrocellulose membrane was treated with a blocking solution (Tris-buffered saline (TBS) containing 0.1% (v/v) Tween 20 and 6% (w/v) non-fat dry milk) to reduce non-specific binding. Immunoreactions were carried out using a rabbit polyclonal IgG GABA_B1_R (Santa Cruz),) or rabbit-derived anti-rat GABA_B1_R antibody (Abcam Limited, UK), PKA-α (Santa Cruz), cytochrome C a rabbit polyclonal (Santa Cruz) and cleaved caspase-3 a rabbit polyclonal antibody recognizing 17-kDa active subunit of caspase-3 (Cell signaling) antibodies (1:1000, 24 h, 4°C). Following rinses, horseradish peroxidase conjugated goat anti-rabbit or rabbit anti-goat (Santa Cruz) IgG-HRP (1:10000, Bio-Rad) was added and incubated for 90 min at room temperature. Immunoreactions were also carried out using β-actin antibody (Santa Cruz) for equal protein as loading controls. Proteins were detected by chemiluminescence using an ECL-detecting reagent (Amersham Pharmacia Biotech, Western blotting detection reagents) according to their protocol and then exposed to X-ray film. The X-ray films were scanned and the optical densities of Western blots were analyzed by densitometry using the computer-based Sigma Gel, version 1.0 (Jandel Scientific, San Rafeal, Chicago, USA).

### Visualization of mitochondrial cytochrome-c release and caspase-3 expression

The in situ analysis of cytochrome-c release and caspase-3 expression was carried out by immunofluorescence technique. Briefly, primary culture of hippocampal neuronal cell (1 × 10^6 ^cells in culture plates) was treated with ethanol, PTZ, ethanol plus PTZ fixed with 4% neutral buffer paraformaldehyde (NBP) and washed with PBS in chilled condition. Cytochrome-c was detected by using mouse anti-cytochrome-c antibody over night at 4°C and rabbit anti-mouse FITC-labeled antibody for 90 min at room temperature (1:250 and 1:100, respectively; Santa Cruz Biotechnology, CA, USA). Subsequently, caspase-3 expression was detected by using rabbit anti-caspase-3 antibody (Cell signaling) over night at 4°C and goat anti-rabbit TRITC labeled antibody (Santa Cruz Biotechnology, CA, USA) for 90 min at room temperature (1:250 and 1:100, respectively) in dark and slides were mounted with Prolong Antifade reagent (Molecular Probes, Eugene, OR, USA). Cytochrome-c (green) and caspase-3 (red) staining patterns were acquired by use of a confocal laser scanning microscope (Fluoview FV 1000, Olympus, Japan).

### Histological analysis and detection of apoptosis

Propidium iodide (PI) and Fluoro-Jade-B staining was performed as previously described [29,30,52]. After the maternal exposure of PTZ (40 mg/kg i.p for 15 days) during pregnancy at GD 17.5 animal were anesthetized by giving sodium pentobarbital (50 mg/g. i.p). Fetus were removed, fixed in cold 4% NBP for 48 h and cryoprotected by immersion in to 20% sucrose phosphate buffer for 48 h at 4°C. Whole fetus were frozen at O.C.T compound (A.O. USA) and 14 μm section were made in the coronal planes (Leica cryostat CM 3050C, Germany). Sections were thaw mounted on the probe-on plus charged slide (Fisher). Slides were dipped in 1 μg/ml of PI solution in PBS for 20 min at room temperature with gentle mixing and washed twice with PBS for 10 min. Glass cover slip were mounted on glass slides with mounting medium. PI filter used to detect the PI staining (Red color) and FITC filter used to detect Fluoro-Jade-B (Green color). For images we used a Zeiss fluorescent microscope (Zeiss, Germany) and confocal microscope (Olympus, Japan). Photographs were taken with a soft imaging systems video camera.

### Data analysis and statistics

The object band from RT-PCR and Western blot were scanned and analyzed by densitometry using a computer based on the Sigma Gel System (SPSS Inc., Chicago, IL). Density values were expressed as mean ± SEM. One-way ANOVA analysis followed by Tukey-Kramer multiple-comparisons test was performed to determine the significance of differences between relevant treatment groups. In every case, the acceptance level for statistical significance was *P < 0.05 and **P < 0.01.

## Abbreviations

PKA-α: Protein kinase A-α; PTZ: pentylenetetrazol; GD: gestational days; CNS: central nervous system; EEG: electroencephalography; FAS: Fetal alcohol syndrome; GABA: gamma-aminobutyric acid; DMEM: Dulbecco's modified Eagle medium; DEPC: diethyl pyrocarbonate; NBP: Neutral buffer paraformaldehyde; FJB: Fluoro-Jade B; PI: propidium iodide.

## Competing interests

The authors declare that they have no competing interests.

## Authors' contributions

MIN designed and conducted experiments; LS oversaw the project together with MOK. All authors read and approved the final manuscript.
